# Continuous Monitoring of Essential Tremor Using a Portable System Based on Smartwatch

**DOI:** 10.3389/fneur.2017.00096

**Published:** 2017-03-15

**Authors:** Xiaochen Zheng, Alba Vieira Campos, Joaquín Ordieres-Meré, Jose Balseiro, Sergio Labrador Marcos, Yolanda Aladro

**Affiliations:** ^1^Department of Industrial Engineering, Technical University of Madrid, Madrid, Spain; ^2^University Hospital of Getafe, Getafe, Madrid, Spain

**Keywords:** essential tremor, smartwatch, accelerometer, continuous monitoring, remote diagnosis

## Abstract

**Introduction:**

Essential tremor (ET) shows amplitude fluctuations throughout the day, presenting challenges in both clinical and treatment monitoring. Tremor severity is currently evaluated by validated rating scales, which only provide a timely and subjective assessment during a clinical visit. Motor sensors have shown favorable performances in quantifying tremor objectively.

**Methods:**

A new highly portable system was used to monitor tremor continuously during daily lives. It consists of a smartwatch with a triaxial accelerometer, a smartphone, and a remote server. An experiment was conducted involving eight ET patients. The average effective data collection time per patient was 26 (±6.05) hours. Fahn–Tolosa–Marin Tremor Rating Scale (FTMTRS) was adopted as the gold standard to classify tremor and to validate the performance of the system. Quantitative analysis of tremor severity on different time scales is validated.

**Results:**

Significant correlations were observed between neurologist’s FTMTRS and patient’s FTMTRS auto-assessment scores (*r* = 0.84; *p* = 0.009), between the device quantitative measures and the scores from the standardized assessments of neurologists (*r* = 0.80; *p* = 0.005) and patient’s auto-evaluation (*r* = 0.97; *p* = 0.032), and between patient’s FTMTRS auto-assessment scores day-to-day (*r* = 0.87; *p* < 0.001). A graphical representation of four patients with different degrees of tremor was presented, and a representative system is proposed to summarize the tremor scoring at different time scales.

**Conclusion:**

This study demonstrates the feasibility of prolonged and continuous monitoring of tremor severity during daily activities by a highly portable non-restrictive system, a useful tool to analyze efficacy and effectiveness of treatment.

## Introduction

Essential tremor (ET), the most prevalent tremor disorder, is a postural and kinetic tremor affecting 4.6–6.3% of adults above the age of 60–65 (1). It represents a family of diseases rather than a single one, which could be associated with gait disorders and cognitive impairments (2). Although ET is not a life-threatening disease, it has an important negative impact on all aspects of life quality, including social and psychological, due to their exacerbation in public (3). It mainly affects the upper limbs with the consequent interference in basic activities of daily living, such as handwriting, dressing, eating, and self-care (4). Progress in the treatment of ET is limited because of the poor understanding of many of the underlying conditions (5). Tremor analysis during daily living is crucial to better understand the patient status and to evaluate treatment effects (6). In clinical practice, it is usually assessed by several tremor validated rating scales (7–12). Although these have demonstrated clinical utility, they require the presence of a clinician for scoring. Their results are subject to clinical judgment and show significant intra- and inter-explorer variabilities. The main limitation is that they only provide an instantaneous, subjective, and qualitative assessment of tremor intensity during a clinical visit and do not allow extended continuous monitoring of tremor fluctuation patterns throughout the day or in home environments (6, 13).

Essential tremor shows a frequency between 4 and 12 Hz and variable amplitude, depending on stress level, position, voluntary movements, and disease evolution (14–17). It is higher than the frequency of normal voluntary human movements. Therefore, it is possible to detect tremor on the basis of the frequency difference (3, 13). The feasibility of classifying tremor according to the acceleration data collected from patient’s wrist has also been reported (18). A variety of tremor data collecting systems based on acceleration sensors have been developed and applied in many studies (6, 19–27). However, most of these systems are designed in the laboratory environment and will cause inconvenience to the patient in case of long-term application. For example, the Kinesia™ (CleveMed) system has shown a good correlation with scale tremor scores, for all, rest, positional, and kinetic tremor (6, 28). Although this system is portable, it still limits the movement of the limb and it is hard to monitor the patient remotely due to the distance limitation. The development of wearable technology, such as smartwatch, smart band, and smart glasses, provides a new method to collect motion data more conveniently. The study presented in Ref. (29) has verified the practicability of using a smartwatch to analyze and diagnose tremor.

This research testifies to the feasibility and potential clinical utility of a new highly portable system capable of continuously recording arm motion data and sending them to a remote server through a mobile terminal in real time. The aim is to quantify the amplitude and frequency of the tremor and establish greater traceability with daily activities. The main device of the system is an Android smartphone and a smartwatch that contains a triaxial accelerometer. There is a database on a remote server, managing all the data collected from the patient without distance limitation. This system is able to monitor the patient’s movement continuously without introducing much inconvenience. Accordingly, a quantitative overall situation of the patient in a long-range scenario is accessible.

## Materials and Methods

This study is approved by the ethics committee of the Getafe University Hospital (Madrid, Spain) and is conducted in accordance with the Declaration of Helsinki of the World Medical Association. All participant patients have signed an informed consent. The data collecting system only collects anonymous data from the smartwatch and the smartphone. Deidentified data were used, so that only the local investigator was aware of the source of the data and could associate them to a specific patient. Collected technical data are stored in a local server within the network of Technical University of Madrid protected by several firewalls. In addition, only authorized researchers of the team can access them. Therefore, patient privacy has been well protected throughout the study.

### Subjects

Nine ET subjects, aged 60–77 years (mean 69.0 ± 6.6 SD, six males and three females), with different levels of ET are included, all of them over the moderate level on Fahn–Tolosa–Marin Tremor Rating Scale (FTMTRS). The data of one patient are destroyed due to hardware problem (Android phone battery) and the data of the remaining eight patients are successfully collected, aged 60–77 years (mean 68.0 ± 6.3 SD, six males and two females). All patients selected showed both, postural and intentional tremor, although the tremor levels are different between patients and in each patient. Five patients were under tremor therapy, two were taking propranolol, another couple of them were on treatment with primidone, and the other one was taking clonazepam. The medication was maintained throughout the study. The data of a healthy male, aged 54 years are also recorded to compare with the data of the patients.

### Monitoring System

A portable human movement monitoring system has been previously developed (30). This three-layer system is composed of a Pebble smartwatch,^1^ which contains a triaxis accelerometer and Bluetooth 4.0, for recording the user’s arm movement data; an Android smartphone for receiving data from Pebble and uploading them to a remote server; and a cross-platform document-oriented NoSQL MongoDB database^2^ on remote server for data storage and analysis. The information collected using this system includes three-axis arm movement acceleration values. The feasibility of analyzing tremor using the data collected from a smartwatch has been validated in our previous work (31).

### Procedures of Data Collection

At the first visit, each patient receives an initial training session to learn how to use the system (smartwatch and Android phone). Then, wearing firmly the watch on his/her wrist more severely affected (on the dominant hand if both are equally affected), the patient realizes the standardized tasks at hospital under the supervision of a neurologist from the research team. The tasks of FTMTRS (12) included are the following: (1) keeping hands relaxed on the lap for evaluating rest tremor, (2) holding their arms extended horizontally for studying postural tremor, and (3) repeating finger–nose test several times, writing “this is a sample of my writing,” signing, dating, drawing two spirals and a line between two points and two bars, and emptying a full glass of water into another empty one several times for examining kinetic and intentional tremor. All tasks are performed during 15 s except writing and drawing. Patients perform the tasks several times until they learn them. Each item is rated on a scale from 0 to 4 corresponding to the severity of tremor by the patient (not induced by the neurologist) and by two neurologists. In order to assure an adequate accomplishment of the standardized tasks at home, patients will have a fully documented guide containing clear instructions on how to perform and quantify each task. When performing the standard tasks, the patients were asked to report a self-evaluation score for each task and the system would add a timestamp for each task, so that the task-induced tremor can be identified by comparing the timestamp. During these tasks, all movement data are being collected and uploaded to the remote server in real time.

The movement monitoring process begins once the patient is able to manage the system and perform the tasks correctly. The outpatient monitoring consists of continuous capturing of all unrestricted movements since waking up in the morning until bedtime at night and during the predefined task performed three times every day. Patients should perform and rate all tasks according to FTMTRS, oriented by a fully documented guide.

### Data Analysis

Three ratings of ET were performed on each patient: the assessment of neurologists during standardized tasks at hospital, the self-assessment of the patients during standardized tasks at home, and the analysis result based on the data collected during continuous monitoring. Since each patient performed standardized tasks three times per day and the monitoring lasts for several days, the average value of the patient’s self-assessment scores of all tests for the same patient is calculated to represent the final score of patient’s self-assessment result. Pearson correlation was used to evaluate the agreement degree between different scores.

#### Tremor Identification

Tremor was identified according to the frequency difference between voluntary movements and ET. From previous studies, it is known that the frequency of ET is between 4 and 12 Hz. The tremor of the patients in this research shows a lower frequency, between 4 and 8 Hz. We used a frequency filter of this range to identify tremor and eliminate intended human actions, whose amplitude is usually higher than tremor. When analyzing data collected from a healthy subject, infrequently we found high frequency activities that are easy to identify according to this difference of amplitude. Fast Fourier Transform (FFT) can be used to transfer the raw acceleration data from time domain to frequency domain. After the transformation, relevant frequencies are filtered. Therefore, the method is to cast the collected data into the frequency domain through FFT and check if amplitudes above certain threshold between 4 and 8 Hz (31). All movement data were collected with a frequency of 25 Hz; therefore, after the FFT operation, the highest reliable frequency was 12.5 Hz. This sampling rate is enough for the analysis of tremor which is lower than 12 Hz. To see details, the reviewer or the readers can consult the original data at https://drive.google.com/file/d/0B3UOl_J6yg0vOU8wcnhQVWlmYkk/view, where some figures show the original acceleration data and corresponding FFT results of the healthy subject and one of the patients.

#### Tremor Classification

The acceleration data collected on regular time basis were transformed into the frequency domain with the FFT operation. After the transformation, the frequencies between 4 and 8 Hz were filtered. Since the amplitude can only reflect the information of each corresponding frequency, the tremor energy in this frequency range was calculated to better represent the complete information.

Suppose *a*(*t*) is the acceleration at the time *t* and *A*(*jw*) is the Fourier transformation of *a*(*t*), then:

$$\overset{\infty }{\underset{-\infty }{\int }}{\left|a\left(t\right)\right|}^{2}dt=\frac{1}{2\pi }\overset{\infty }{\underset{-\infty }{\int }}{\left|A\left(jw\right)\right|}^{2}dw$$

where ${\left|A\left(jw\right)\right|}^{2}$ is the power spectral density of the acceleration *a*(*t*).

The relevant tremor energy becomes

$$E=\frac{1}{2\pi }\overset{W_{U}}{\underset{W_{L}}{\int }}{\left|A\left(jw\right)\right|}^{2}dw$$

*W_L_* and *W_U_* are the tremor frequency range, which are 4 and 8 Hz, respectively. And *E* is the energy assigned to the ET relevant frequencies for the specific period of time.

Tremor was classified into four levels corresponding to the FTMS scale according to the energy of the tremor in the frequency domain. The basic analysis unit in this research is 1 min; 1,500 records were included in each basic analysis unit and around 160 values locate in the 4–8 Hz frequency range. The data collected in 1 h from four patients (with tremor grades 1–4, respectively, according to the evaluation of the neurologists) were adopted to define the thresholds.

**Figure 1:**
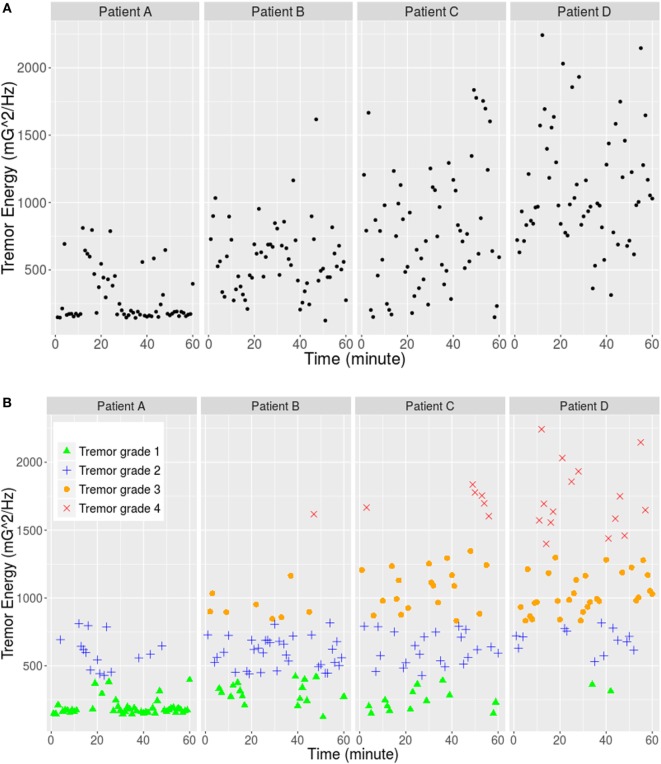
**(A)** Tremor energy between frequency range 4 and 8 Hz of each minute for four patients. **(B)** K-mean clustering result: the data were separated into four classes corresponding to the four tremor grades of FTMS.

First, FFT operation was applied and the tremor energy was calculated for each minute (60 values for each patient and 240 values in total) as it is shown in Figure 1A. Second, the 240 energy values were classified into four groups using the K-mean clustering method, and the result is explained in Table 1 and Figure 1B.

**Table 1:** **K-mean clustering result of the tremor energy based on the data collected from four patients**.

	Cluster 1	Cluster 2	Cluster 3	Cluster 4
Cluster size	77	84	57	22
Cluster means	229.30	620.36	1,036.89	1,722.60
Max value	421.03	816.34	1,004.71	2,243.06
Min value	124.05	429.54	993.42	1,398.99

Then, the thresholds are defined as below:

$${\text{Th}}_{i}=0.5*\left\{\text{Max(Cluster }i\text{)}+\text{Min}\left[\left(\text{Cluster }i+1\right)\right]\right\}(i=1,2,3)$$

The results are rounded to integers as the three thresholds, which are 425, 904, and 1,202 (mG^2^), respectively. They classify the tremor into four levels (“Light,” “Medium,” “High,” and “Very high”) corresponding to grades 1–4 of FTMTRS, respectively. The FTMTRS scores of the neurological rating obtained during standardized assessments at hospital were used as a gold standard, and the mean FTMTRS scores of all six tasks were employed for correlation analysis (Pearson correlation, *r* = 0.80; *p* = 0.005). After this procedure, tremor condition of 1 min can be represented with a single quantitative value. By combining the analysis result of every minute, the tremor condition in longer period can be revealed, for instance, in each hour or each day.

#### Data Generalization

Since the proposed data collecting system is able to collect the daily movement data of the patients continuously, a larger scale analysis covering a longer period is possible. As the ET energy is not constant in time, it is convenient to provide a time independent yet consistent method to present the ET level. By using this method, it will be possible to compare different episodes for the same patient or different patient results. The proposed method represents a bi-dimensional diagram where on the *Y* axis represents the maximum energy value, $\underset{i\epsilon [1,..,N]}{\text{max}}(E(i))$, where *N* is the number of minutes under consideration and *E*(*i*) is the energy associated with every minute. On the *X* axis, the averaged energy $\overset{N}{\underset{i=1}{\sum }}E(i)/N$ is represented, in such a way that the structure of this chart remembers the Receiver Operating Characteristic diagram. High *Y* values and low *X* values or low *Y* values and high *X* values mean low ET intensity. Therefore, it is feasible to use the area of the rectangle defined by (0, 0) and the point representing the patient data as the ET intensity metrics. Thus, tremor situation of every hour can be represented with a single point, then, the analysis scale can be enlarged by 60 times.

## Results

All patients wore the smartwatch consistently and performed the standardized tasks as required every day. Each patient wore the smartwatch 3 days at least and the data of 2 days from each of them were selected for the analysis. More details about the data collected from the eight patients are shown in Table 2.

**Table 2:** **Summary of the data collected from the eight patients**.

Item	Value
Total number of patients	8
Total number of hours (days)	208 (16 days)
Hours per patient (minimum)	17
Hours per patient (maximum)	34
Hours per patient (mean)	26
Hours per patient (SD)	6.05

**Figure 2:**
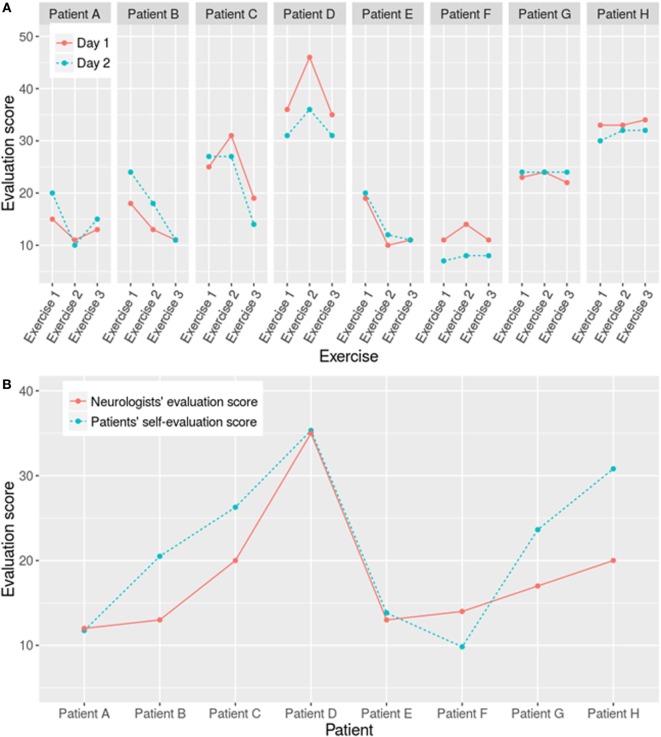
**(A)** Day-to-day correlation between patients’ auto-assessment scores during 2-day standardized tasks and, **(B)** Correlation of standardized tasks between auto-assessment and neurologist scores, of the all eight patients.

Patient’s self-assessment scores between tremor ratings from days 1 and 2 for each patient show good agreement (*r* = 0.87, 95% CI: 0.72−0.94, *p* < 0.001) (Figure 2A). Significant correlation is also obtained between neurologist’s FTMTRS and patient’s FTMTRS mean auto-assessment scores (*r* = 0.84, 95% CI: 0.33−0.97, *p* = 0.009) (Figure 2B), both scores of all patients are listed in Table 3.

**Table 3:** **Rating scores of the patient’s self-assessment and neurologists’ assessment**.

Patient no.	Mean Fahn–Tolosa–Marin Tremor Rating Scale scoreAll test, all days; patient’s auto-assessment	Neurologist score
A	23.63 (±0.92)	17
B	35.33 (±5.61)	35
C	20.50 (±6.32)	13
D	9.83 (±2.64)	14
E	13.83 (±4.45)	13
F	26.28 (±5.11)	20
G	11.75 (±5.05)	12
H	30.81 (±1.36)	20

**Figure 3:**
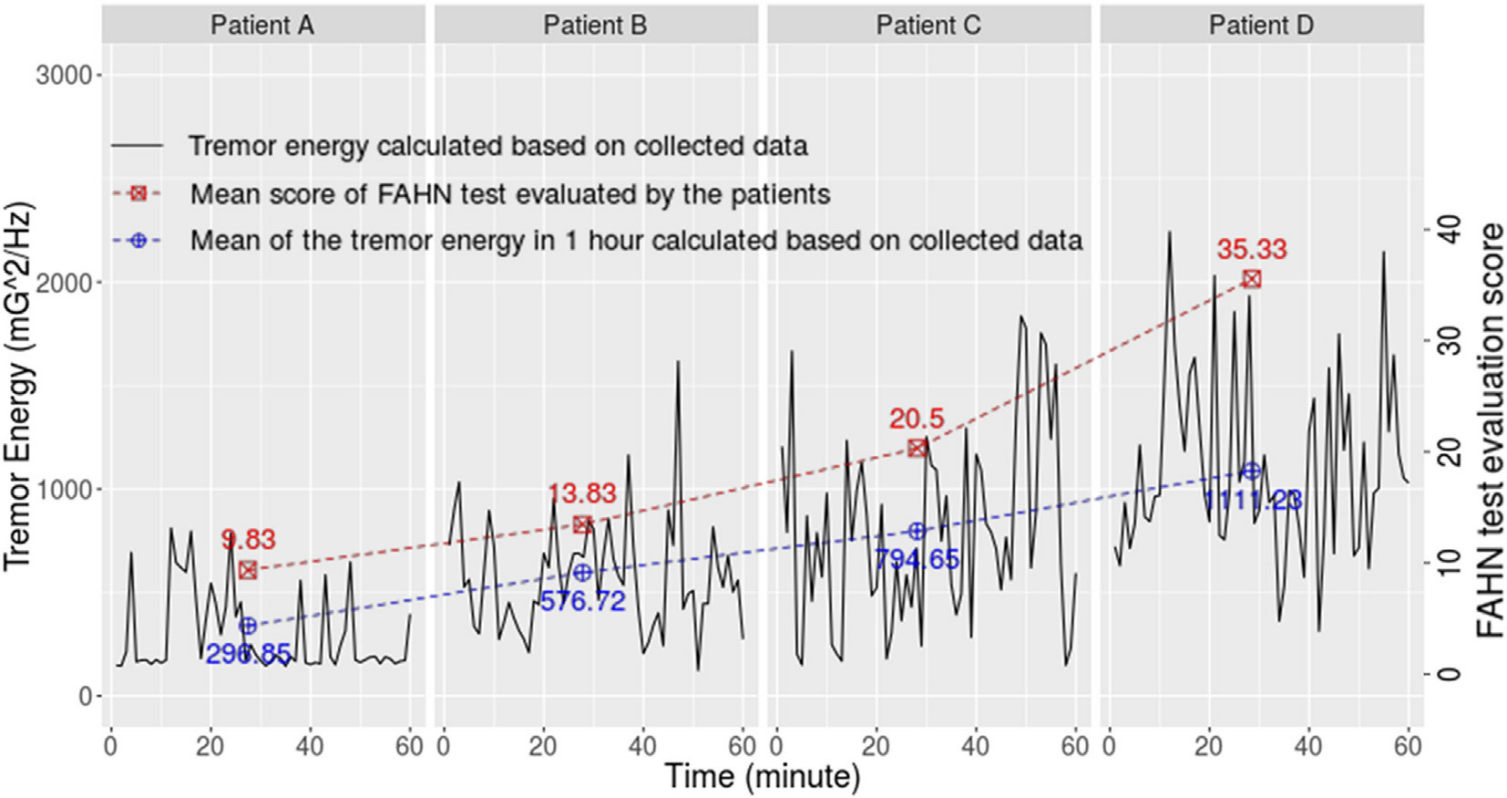
**Correlation between results based on collected data and the auto-assessment scores reported by four patients with different tremor degrees**.

The data of four patients and the healthy subject were further analyzed considering their tremor levels and data integrity (each represents a tremor level). Their information is shown in Table 4. Figure 3 exhibits the validation result using the similar method as Pulliam et al. (28). The correlation between the patients’ self-assessment score and the calculation result based on the collected data is significant (*r* = 0.97, 95% CI: 0.11−0.99, *p* = 0.032).

**Table 4:** **Basic information of the patients and healthy subject involved in the experiment**.

Subject	Sex	Age	Tremor grade	Arm with higher tremor
Healthy subject	Male	54	0	None
Patient A	Male	68	1	Right
Patient B	Male	72	2	Right
Patient C	Male	75	3	Left
Patient D	Male	60	4	Equal

**Figure 4:**
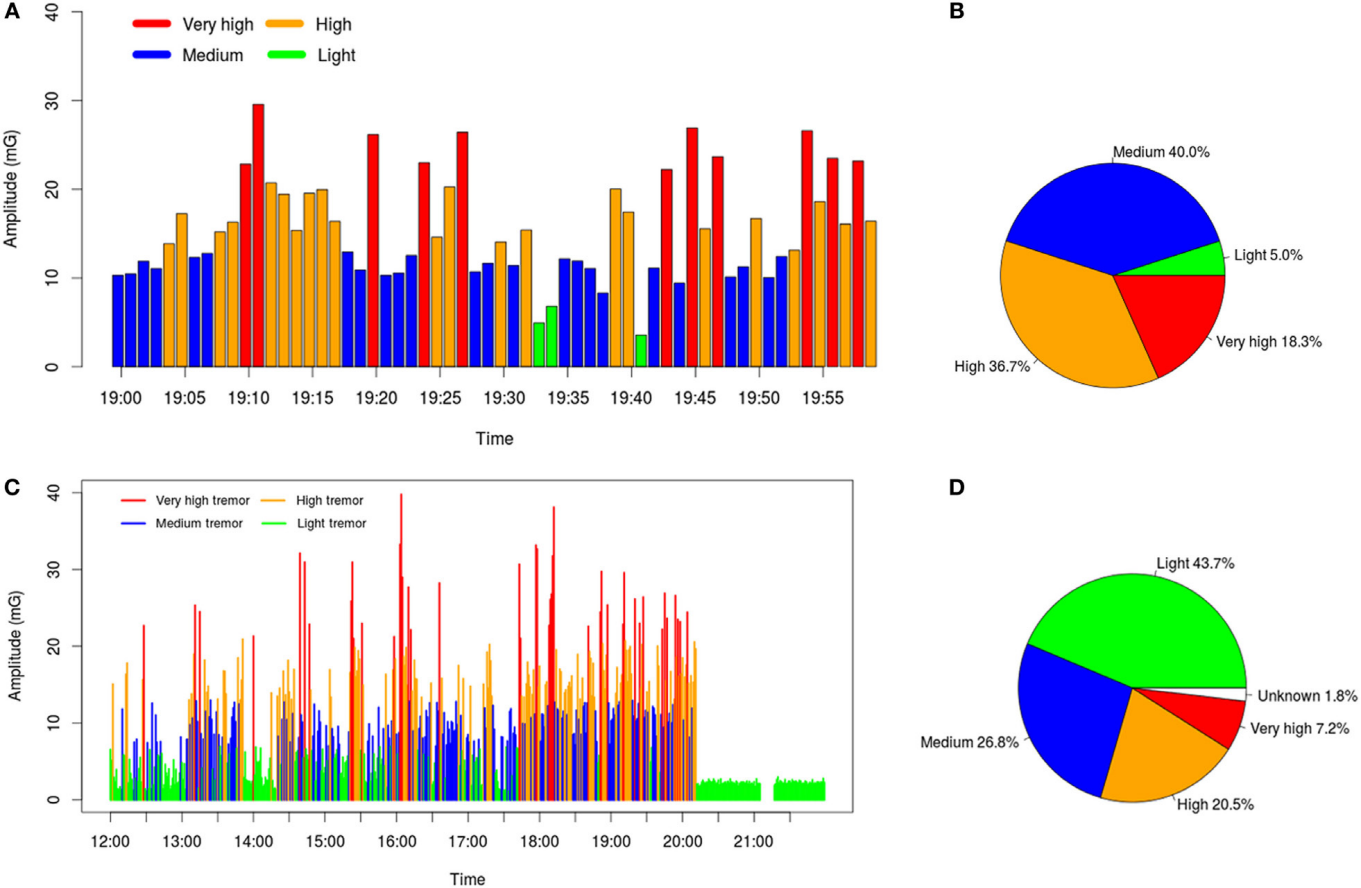
**(A)** Tremor severities of every minute in an hour (19:00–19:59). **(B)** Percent of each level of tremor during this hour: high- and very high-level tremor appears in more than 65% of the overall time. **(C,D)** illustrate the collected data in a longer range, which better reflects the overall situation of the patient during 8 h (50% of the day awake). The “Unknown” part means the data during this period are failed to be recorded.

Patient’s tremor in each minute was classified according to the predefined thresholds. The general situation during long-term daily lives was summarized by combining the classification result of every minute. A graphical representation of patient D’s tremor (severe ET) during different time periods is illustrated in Figure 4. This analysis allows not only quantifying the percentage of time in a day with different levels of tremor severity but also querying the moments with more serious tremor (in this case between 18 and 20 h), a potential tool to optimize treatment.

**Figure 5:**
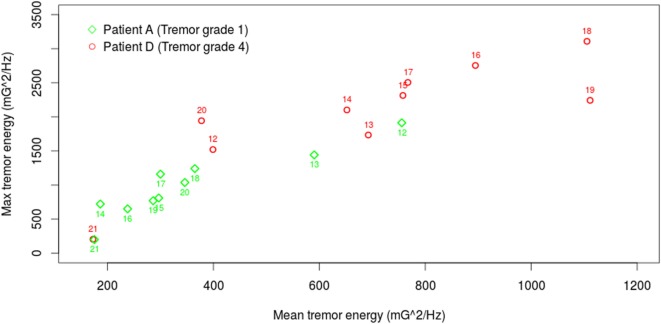
**Daily analysis of tremor rating of patients A and D, who have the lowest and the highest tremor severity, respectively**.

Finally, Figure 5 presents the daily (10 h) tremor ratings of Patient A and Patient D (with the lowest and the highest tremor severity, respectively). The vertical and horizontal components of each point indicate the max and the mean tremor energy value during this hour, respectively. The distribution of patient A’s tremor is closer to the bottom left corner with mean energy less than 400 (mG)^2^/Hz and max energy less than 1,500 except 2 h at noon (12 and 13 o’clock). In contrast, patient D’s tremor ratings are closer to the top right corner at the most of the time.

## Discussion

This work evaluates the feasibility of continuously monitoring the tremor status of ET patients by using a highly portable system, which is based on a smartwatch and a smartphone. With the data collected with this system, hypothetically it would be able to trace quantitatively the progression of the disease in the long term, as well as to measure the effect of different treatments more accurately. The major temporal resolution of tremor status obtained could have great potential in clinical practice and research.

Although we have used for the first time FTMTRS as a gold standard to compare its ratings with the accelerometer’s quantitative measures in ET, this scale includes tasks for postural and kinetic tremor which have demonstrated good correlation with data of motor sensors (9). It has also shown good reliability for evaluating upper extremities tremor, and finally, its logarithmic relationship with tremor amplitude, measured with transducers, has been well documented (32). Since the tasks to measure the intensity of tremor are easy to learn, we have contemplated that patients make their standardized auto-assessment of the severity tremor in order to use the results in our analysis. The correlation among all tremor assessments—from the neurologists, the patients, and the system—has been robust with minimum deviation, being this finding a push for starting to consider auto-assessment as a valid measure, which could avoid videotaping tremor tasks and subsequent ratings by physicians. In agreement with Pulliam et al. (28), the strong relationship between continuous monitoring and standardized assessment would make possible to dispense with the latest in clinical practice.

### System Advantages

This system is highly portable and comfortable. Patients only need to wear the smartwatch and carry the smartphone with them. This will not introduce extra inconvenience, since both devices are very common daily supplies. The communication between the watch and the smartphone is through Bluetooth, allowing the patient to carry the smartphone anywhere around. The data uploading is supported by both Wi-Fi and 3G/4G telephone services, and it is temporarily stored in the smartphone in case that there is no signal, in which case it is later uploaded once Internet is available. The data are managed by an online database, which is accessible remotely. Clinicians are able to trace the situation of the patients at any time through Internet, no matter where the patients are. Moreover, the cost of the system is very low—the main cost is the smartwatch—and it is specially designed for aged people.

### Clinical Application

Compared with the traditional tremor rating scales, this system provides a more accurate quantitative evaluation of the tremor severity for ET patients. It eliminates the bias produced due to the subjective judgment of the neurologist and the patient. Since there is no distance limitation for this system, it is possible for the neurologists to monitor a patient remotely.

The long-term and unrestricted tremor analysis could be a potentially useful tool to determine the fluctuation patterns of ET caused by voluntary movements and other internal or external factors (alcohol ingestion, stress, etc.), as well as to improve treatment efficacy and effectiveness. Regarding the first point, tremor amplitude fluctuations in ET have been reported around 30–50% between assessments using rating scales (33). On the other hand, evidences with sensor motor monitoring are so far scarce and inconclusive; it was reported a 23% variability in an objective assessment performed with a motor sensor system (34), in which these fluctuations were statically associated with high amplitude tremor. However, other authors have not found great amplitude variability in ET patients with continuous monitoring (28). Therefore, the objective assessment and continuous monitoring with accelerometers could be to contribute to know better the nature of this tremor. With respect to the optimization of the treatment, given that clinicians can objectively distinguish and quantify percentages of time throughout the day with different grades of tremor severity, it would be possible to monitor more accurately the effects of the treatment applied. Objective and continuous register are necessary to evaluate the treatment interventions in clinical trial.

### Limitations

In this study, the background activities when tremor appears during the patients’ daily lives were not analyzed. An applet was installed in the smartphone to help patients to record their activities and the feeling of tremor intensity in that moment, but they forgot to record frequently. Therefore, we did not include these data in this study. Current effort is making to simplify the applet and to introduce some alert to remind patients of this task. Knowing the background activities will permit a better traceability of tremor.

This system collects the movement data of patient’s arm. However, tremor of many ET patients also appears in head and legs. In these situations, the data collected with this system cannot accurately reflect the severity of the disease. Further development is necessary to connect more wearable devices into the system.

## Author Contributions

YA and JO-M conceptualized the idea for this study and organized the project. XZ, AVC, JO-M, and YA were involved in the data collection. XZ and JO-M were responsible for the data analysis, and the analysis results were reviewed and critiqued by YA, JO-M, and JB. The patients were managed and evaluated by AVC, YA, JB, and SLM. The final manuscript was written by XZ and YA and was reviewed and improved by JO-M and JB.

## Conflict of Interest Statement

The authors declare that the research was conducted in the absence of any commercial or financial relationships that could be construed as a potential conflict of interest.

## Footnotes

^1^https://www.pebble.com/pebble-smartwatch-features (Updated May 2016).

^2^https://www.mongodb.com/ (Updated May 2016).
